# A remarkable new species of *Alloscorpiops* Vachon, 1980 from a cave in Vietnam (Scorpiones, Euscorpiidae, Scorpiopinae)

**DOI:** 10.3897/zookeys.500.9421

**Published:** 2015-04-27

**Authors:** Wilson R. Lourenço, Dinh-Sac Pham

**Affiliations:** 1 Muséum national d’Histoire naturelle, Département Systématique et Evolution, UMR7205, CP 053, 57 rue Cuvier, 75005 Paris, France; 2 Institute of Ecology and Biological Resources (IEBR), Vietnam Academy of Science and Technology (VAST), 18 Hoang Quoc Viet, Cau Giay, Hanoi, Vietnam

**Keywords:** Scorpion, Scorpiopinae, *Alloscorpiops*, new species, Vietnam

## Abstract

Among the genera of the subfamily Scorpiopinae Kraepelin, 1905 *Alloscorpiops* remains rather discrete. Only recently new species were added to this genus, increasing its number from two to five. Therefore, species of *Alloscorpiops* remain rare. One remarkable new species, *Alloscorpiops troglodytes* **sp. n.**, is described on the basis of a single male specimen collected inside a cave from Song Thanh Nature Reserve, Cha Vanh Commune, Nam Giang District in Vietnam. The new species presents most features exhibited by scorpions of the genus *Alloscorpiops*, but it is characterized by reduced size, slender body and elongated pedipalps. This new scorpion taxon represents the third species of Scorpiopinae discovered in a cave system, and may be another endemic element in the fauna of Vietnam.

## Introduction

In his revision of the genus *Scorpiops*, Vachon (1980) described three new subgenera, *Alloscorpiops*, *Euscorpiops*, and *Neoscorpiops*, in addition to the nominotypical subgenus *Scorpiops*. *Alloscorpiops* was defined on the basis of an important ‘majorante’ neobothriotaxy with 10–12 ventral trichobothria on the surface of pedipalp chela-hand, whereas the other subgenera presented only four trichobothria. Vachon (1980) assigned two species to this subgenus: Scorpiops (Alloscorpiops) anthracinus Simon, 1887 (as type species of the subgenus) and Scorpiops (Alloscorpiops) lindstroemii Thorell, 1889.

Stockwell (1989), in an unpublished thesis dissertation, proposed raising all the subgenera within the family Scorpiopidae to the rank of genera; however, his proposition could not be validated since his dissertation was never published. Finally, Lourenço (1998) confirmed this decision. The four subgenera were elevated to generic rank and the monotypic genera *Parascorpiops* Banks, 1928 and *Dasyscorpiops* Vachon, 1974 were added, thus bringing the total number of genera to six.

In the present note, a remarkable new species belonging to the genus *Alloscorpiops* is described from a cave in Song Thanh Nature Reserve, Cha Vanh Commune, Nam Giang District in Central Vietnam. This new scorpion taxon is the third species of Scorpiopinae (Lourenço and Pham 2013, 2014) to be discovered in a cave system and the first one belonging to the genus *Alloscorpiops*. It may be yet another endemic element in the fauna of this country.

### Present composition of the genus *Alloscorpiops* Vachon, 1980

Alloscorpiops (Alloscorpiops) anthracinus (Simon, 1887), Myanmar

Alloscorpiops (Alloscorpiops) lindstroemii (Thorell, 1889), Myanmar

Alloscorpiops (Laoscorpiops) calmonti Lourenço, 2013, Laos

Alloscorpiops (Alloscorpiops) citadelle Kovařík, 2013, Thailand

Alloscorpiops (Alloscorpiops) wongpromi Kovařík, Soleglad & Košulič, 2013, Laos, Thailand

Alloscorpiops (Alloscorpiops) troglodytes sp. n., Vietnam

The species *Alloscorpiops lindstroemii* (Thorell, 1889) was considered a synonym of *Alloscorpiops anthracinus* (Simon, 1887) by Kovařík (2013). This decision is, as usual, sustained mainly by personal speculation without the examination of the type material of both species (see also Lourenço 2013; Kovařík et al. 2013; Lourenço and Pham 2015). Inversely, Vachon (1980; in litt.) did examine the types of these species and found some differences which led him to consider them as valid. Some of these characters are expressed herein (key presented after the description). In absence of more solid evidence to validate this synonymy, *Alloscorpiops lindstroemii* (Thorell, 1889) is restored at present.

## Methods

Illustrations and measurements were produced using a Wild M5 stereo-microscope with a drawing tube and an ocular micrometer. Measurements follow Stahnke (1970) and are given in mm. Trichobothrial notations follow Vachon (1974, 1980) and morphological terminology mostly follows Vachon (1952) and Hjelle (1990).

## Taxonomic treatment

### Family Euscorpiidae Laurie, 1896 Subfamily Scorpiopinae Kraepelin, 1905

#### Genus Alloscorpiops

Taxon classificationAnimaliaScorpionesEuscorpiidae

Vachon, 1980

##### Diagnosis of the new species.

The new species shows several of the characteristics already defined for the genus *Alloscorpiops* (Vachon 1980; Soleglad and Sissom 2001). It presents, however, a small size relative to other species of the genus, male 20.9 mm in total length and a very pale yellow coloration. The new species is characterized by the trichobothrial patterns of some ‘territories’ or series. Femur with three trichobothria: dorsal, internal and external. Patella with two dorsal, one internal, 14 ventral and only 21 external trichobothria. Chela-hand with an unusual number of 9 ventral trichobothria, two dorsal (**Dt**, **Db**), two internal (**ib**, **it**), **Est**, five **Et**, **Esb** and three trichobothria in the **Eb** series. The annular ring is very weakly marked. Pectines with 9-9 teeth and absence of fulcra.

#### Description of the new species

##### Alloscorpiops (Alloscorpiops) troglodytes sp. n.

Taxon classificationAnimaliaScorpionesEuscorpiidae

http://zoobank.org/299E145C-F085-4012-B8D4-B66EBBF7A616

Figs 1–6 , 7–13

###### Type materials.

Vietnam, Song Thanh Nature Reserve, Cha Vanh Commune, Nam Giang District, inside cave (Fig. 14), approximately 60 m from entry, 20/XII/1958 (B. Dejenböl). Male Holotype. Deposited in the Muséum national d’Histoire naturelle, Paris.

**Figures 1–6.**
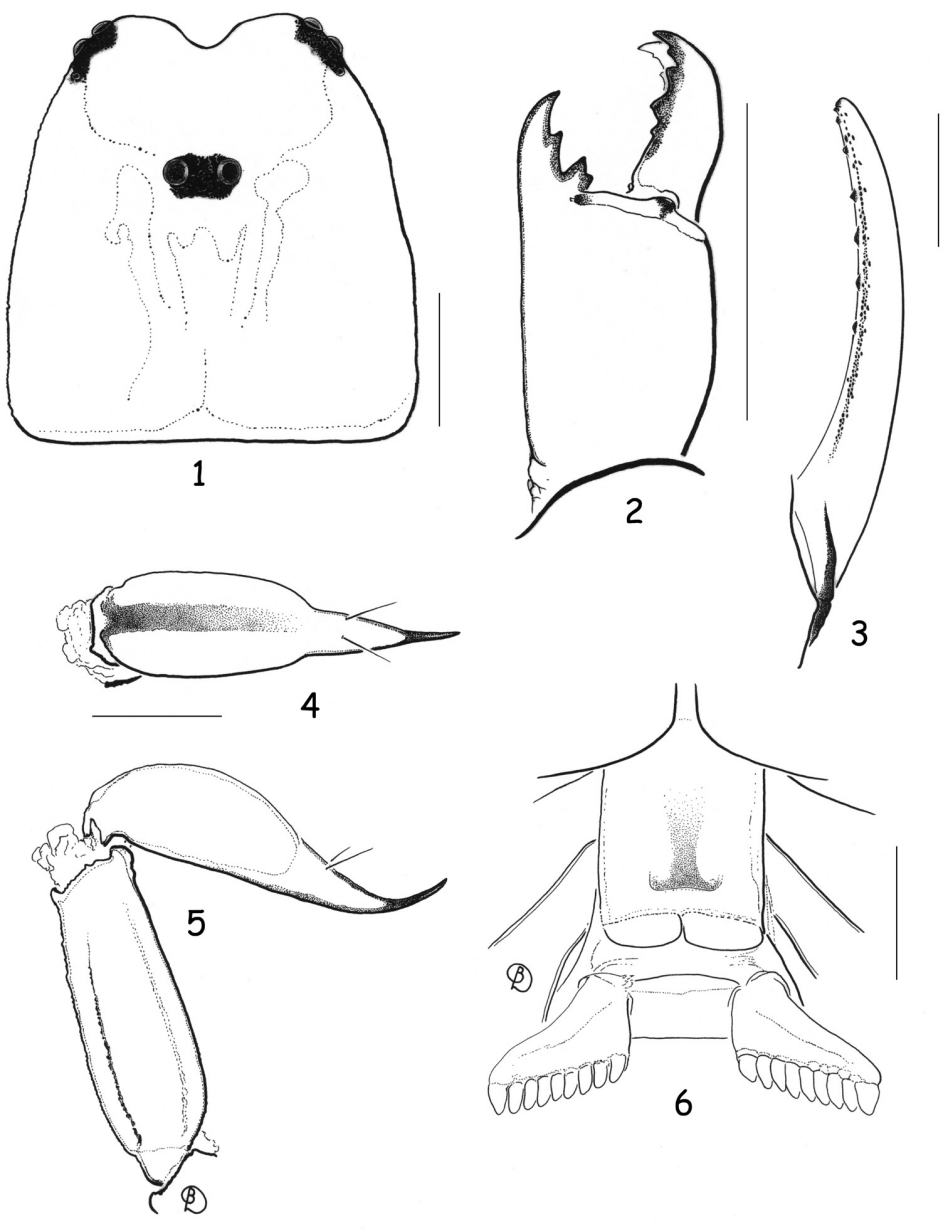
*Alloscorpiops troglodytes* sp. n. Male holotype. **1** Carapace **2** Chelicera, dorsal aspect **3** Cutting edge of movable finger with rows of granules **4** Telson, ventral aspect **5** Metasomal segment V and telson, lateral aspect **6** Ventral aspect, showing sternum, genital operculum, and pectines. Scale bars: 1 mm.

**Figures 7–13.**
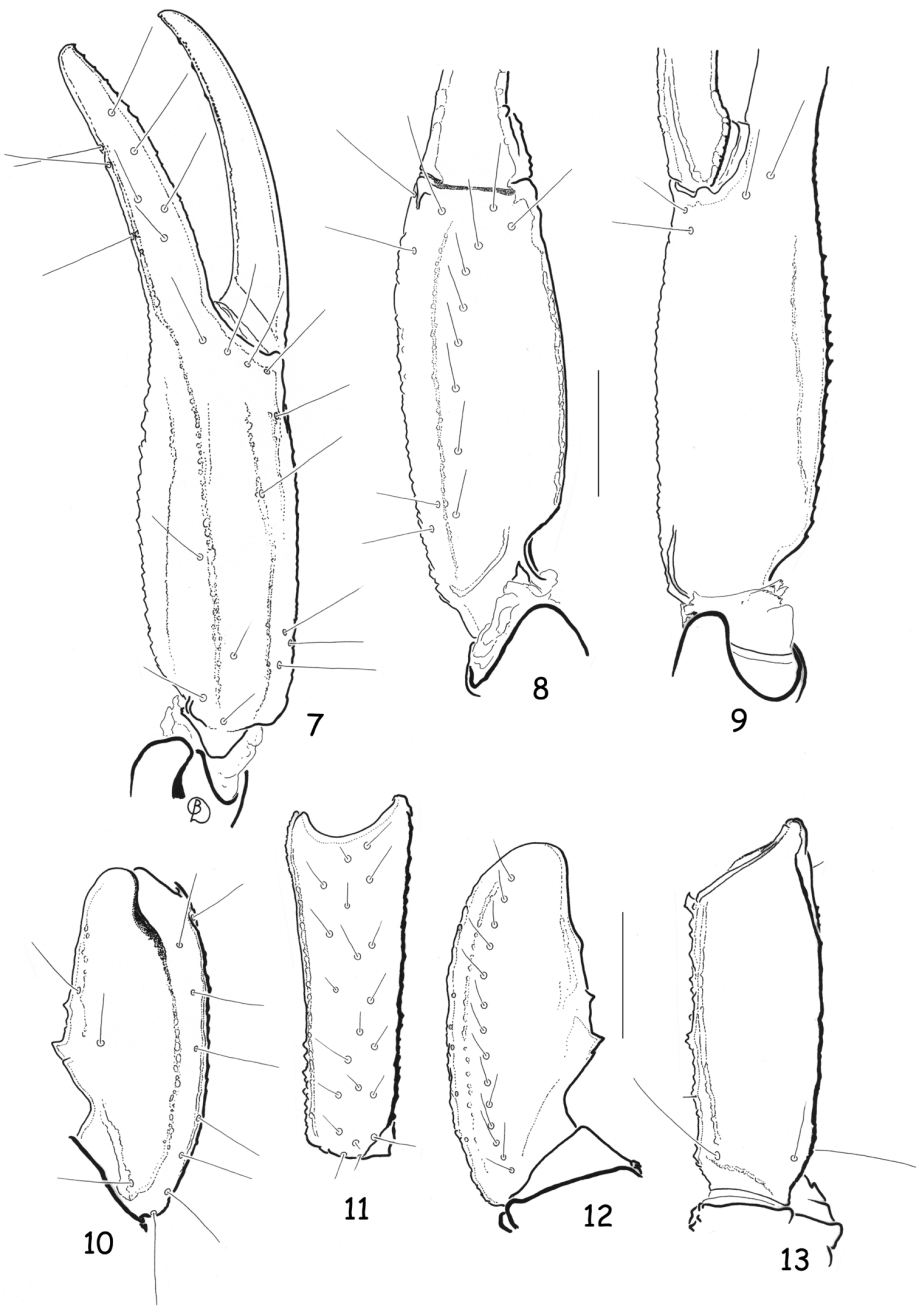
*Alloscorpiops troglodytes* sp. n. Male holotype. Trichobothrial pattern **7–9** Chela, dorso-external, ventral and internal aspects **10–12** Patella, dorsal, external and ventral aspects **13** Femur, dorsal aspect. Scale bars: 1 mm.

**Figure 14.**
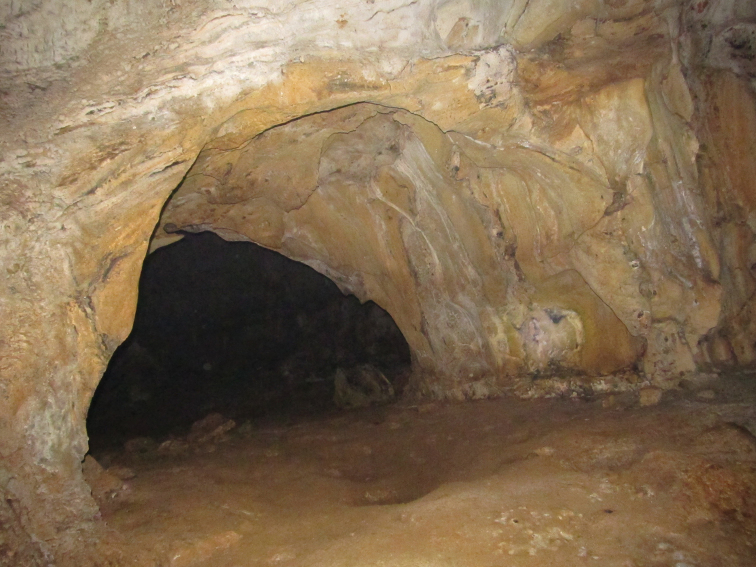
A typical cave of the Song Thanh Nature Reserve cave system. View of the entrance. [Photo courtesy of N.Q. Truong]

###### Etymology.

The specific name refers to the natural habitat where the new species was found.

###### Description.

The general coloration is yellow to pale yellow. Carapace and tergites yellow. Metasomal segments yellow to pale yellow; telson yellow; base of aculeus yellow and tip slightly reddish. Chelicerae yellow without spots; teeth slightly reddish. Pedipalps yellow; chela fingers slightly red. Legs pale yellow. Venter pale yellow; pectines totally pale, almost white.

*Morphology.* Carapace weakly granular, furrows moderately deep. Median eyes anterior to centre of carapace; three pairs of lateral eyes, the third pair only slightly smaller than the first two. Sternum pentagonal, longer than wide. Tergites weakly granulated, almost smooth; VII with four weakly marked carinae. Pectinal tooth count 9-9; fulcra absent. Sternites smooth and shiny; VII with four vestigial carinae and some punctations. Metasomal segment I wider than long; segment II as long as wide; segments III to V longer than wide; 10-8-8-8-7 carinae present on segments I to V, weakly marked; dorsal carinae on segments I–IV with a single, weakly marked posterior spinoid granule; metasomal tegument very weakly granulated almost smooth; ventral carina on segment V without spinoid granules. Telson vesicle totally smooth. Pedipalps: femur with dorsal internal, dorsal external, ventral internal and ventral external carinae moderately marked; tegument weakly granular. Patella with dorsal internal, ventral internal, dorsal external, ventral external and external carinae moderately marked; two/three inconspicuous spinoid granules present on internal aspect, the interno-ventral being slightly larger than the interno-dorsal granule; tegument weakly granular. Chela with dorsal marginal, external secondary, ventral internal and ventral carinae moderately to strongly marked; other carinae moderately to weakly marked; tegument granulated dorsally and ventrally. Chelal fingers with two longitudinal series of granules, almost fused, and a few inner accessory granules. Chelicerae dentition as in figure 2 (Vachon 1963); five/six teeth on ventro-internal face of movable finger. Trichobothriotaxy type **C**, as in figures 7–13 (Vachon 1974): see diagnosis.

*Morphometric values (in mm) of male holotype*. Total length (including telson) 20.9. Carapace: length 3.2; anterior width 2.1; posterior width 3.3. Mesosoma length 7.4. Metasomal segment I: length 0.9, width 1.2; II: length 1.1, width 1.1; III: length 1.2, width 1.0; IV: length 1.4, width 0.9; V: length 2.3, width 0.8, depth 0.7. Telson length 2.9. Vesicle: width 0.8, depth 0.8. Pedipalp: femur length 3.1, width 1.2; patella length 2.7, width 1.3; chela length 6.1, width 1.3, depth 1.2; movable finger length 3.1.

#### Simplified key to the species of *Alloscorpiops*

**Table:** 

1	Chela of pedipalp with 3 trichobothria on the **Eb** series	**2**
–	Chela of pedipalp with 5 trichobothria on the **Eb** series	**Alloscorpiops (Laoscorpiops) calmonti**
2	Chela of pedipalp with 10 to 13 ventral trichobothria; patella with 15 to 22 ventral trichobothria	**3**
–	Chela of pedipalp with 9 ventral trichobothria; patella with 14 ventral trichobothria	***Alloscorpiops troglodytes* sp. n.**
3	Patella of pedipalp with 15–16 ventral and 23–25 external trichobothria	**4**
–	Patella of pedipalp with 19–21 ventral and 29–37 external trichobothria	**5**
4	Patella of pedipalp with 16 ventral and 23 external trichobothria	***Alloscorpiops anthracinus***
–	Patella of pedipalp with 15 ventral and 25 external trichobothria	***Alloscorpiops lindstroemii***
5	Patella of pedipalp with 19-21 ventral and 29-34 external trichobothria	***Alloscorpiops citadelle***
–	Patella of pedipalp with 21-22 ventral and 33-37 external trichobothria	***Alloscorpiops wongpromi***

### Ecological aspects of Nam Giang district and Song Thanh Nature Reserve

**Figure 15.**
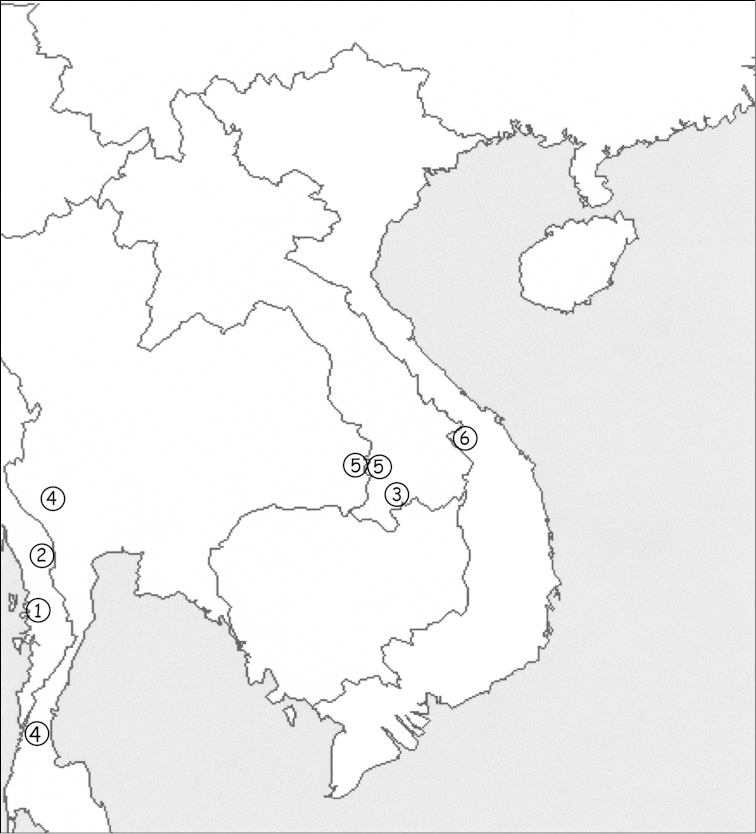
Map of southeast Asia showing the known distribution of the species belonging to the genus *Alloscorpiops*: *Alloscorpiops anthracinus* (**1**), *Alloscorpiops lindstroemii* (**2**), *Alloscorpiops calmonti* (**3**), *Alloscorpiops citadelle* (**4**), *Alloscorpiops wongpromi* (**5**) and *Alloscorpiops troglodytes* sp. n. (**6**).

Cha Vanh commune is located in Nam Giang District, within Song Thanh Nature Reserve in Quang Nam Province along the Vietnam/Laos border. Nam Giang has one of the largest areas of tropical forest in Vietnam and is situated at the intersection of several biogeographical sectors (Fig. 15).

Nam Giang is located within coordinates 15°13’ to 15°41’ N and 107°21’ to 107°50’ E. In the north, it borders highway 14D, which runs from east to west between Thanh My and Dak Oc, along the Vietnam and Laos border. In the south, it connects with Kontum Province at the crest of Lo Xo Mountain and the highway 14D. To the west, it is bordered by Laos PDR and to the east by the waterways of the Thanh and Cai rivers. The altitude of the area ranges from 80 to 2,032 metres above sea level (m.a.s.l.).

This region is part of the central coastal climate zone. With an average temperature of 24.6 °C and a minimum temperature of 20 °C, the weather is hot in comparison with northern Vietnam. The rainy season in the area arrives two to three months after the rainy season north of the Truong Son Mountain range. It ranges from August to December/January, with the most intensive rainfall season occurring between September and November. The dry season coincides with a hot, dry western wind, which speeds up the evaporation process, reduces the humidity, and has a negative impact on the floristic composition of the forest.

There are three main soil types in the area: ferralite humus on rocky mountains (49.7 per cent), typical ferralite in low hill areas (48.9 per cent) and alluvial soils in valleys.

**Figure 16.**
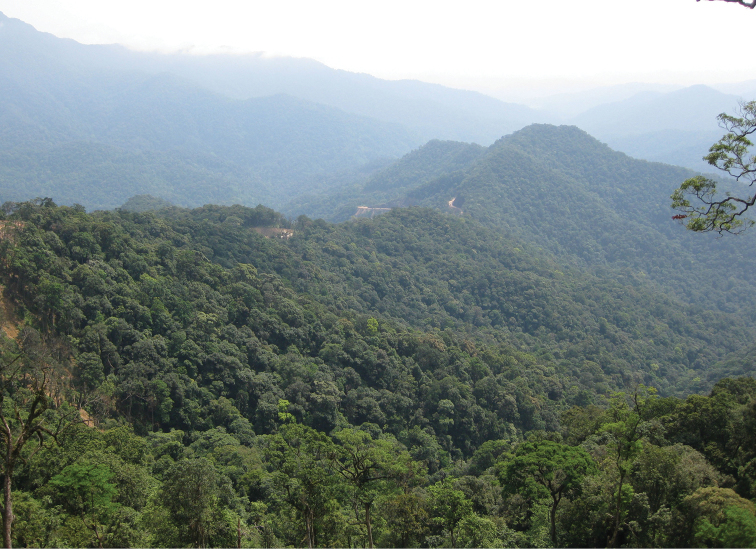
Natural habitat of the new species, *Alloscorpiops troglodytes* sp. n., covered by evergreen forests in Song Thanh Nature Reserve region in Central Vietnam. [Photo courtesy of N.Q. Truong]

The area of Nam Giang is an important component of the Priority Central Truong Son landscape of the Truong Son ecosystem. A rich biodiversity and high numbers of endemic species makes Nam Giang one of the high-priority biodiversity areas in Vietnam. The fauna and flora of Nam Giang is diverse and 95% of Nam Giang is covered by evergreen forests (Fig. 16). Nevertheless the total inventory work on the biodiversity of this region is far from being complete, and many new taxa can be expected to be found during future inventories.

## Supplementary Material

XML Treatment for Alloscorpiops

XML Treatment for Alloscorpiops (Alloscorpiops) troglodytes
